# Translation and cross-cultural adaptation of the functional mobility scale in children with cerebral palsy into Arabic

**DOI:** 10.3389/fpubh.2023.1199337

**Published:** 2023-08-14

**Authors:** Abdulaziz A. Albalwi, Maysoun N. Saleh, Ahmad A. Alharbi, Qais Al-Bakri, Salem F. Alatawi

**Affiliations:** ^1^Department of Physical Therapy, Faculty of Applied Medical Sciences, University of Tabuk, Tabuk, Saudi Arabia; ^2^Department of Physiotherapy, School of Rehabilitation Sciences, The University of Jordan, Amman, Jordan; ^3^School of Medicine, The University of Jordan, Amman, Jordan

**Keywords:** cerebral palsy, functional mobility, FMS, translation, Arabic, Saudi Arabia

## Abstract

**Introduction:**

Cerebral palsy (CP) is a lifelong disorder of posture and movement which often leads to a myriad of limitations in functional mobility. The Functional Mobility Scale (FMS) is a parent-report measure of functional mobility for children with CP at three different distances (5 m, 50 m, and 500 m). This is a cross-sectional study which sought to translate and culturally adapt the FMS into Arabic and to validate the translated version. Functional mobility for children and adolescents with CP in Saudi Arabia was examined.

**Methods:**

The translation methodology complied with the World Health Organization Disability Assessment Schedule 2.0 translation package. A total of 154 children with CP were recruited (mean age 8.16 ± 3.32 years). Parents were interviewed to rate the usual walking ability of their children on the Arabic FMS. The re-test assessment was done with 34 families. The mean time interval between the first and second sessions was 14.3 days (SD = 8.5), with a range of 6–37 days.

**Results:**

Concurrent validity was explored using Spearman’s rank correlation coefficient between scores of the Arabic FMS with their corresponding score on the Gross Motor Function Classification System (GMFCS). Spearman’s *r* values ranged between (−0.895 and **–**0.779), indicating strong to very strong correlations. The Test–retest reliability was examined using Cohen’s weighted kappa, which showed almost perfect agreements. There was greater limitation for functional mobility at longer distances as 55.2% of children could not complete 500 meters (FMS score *N*). Overall, there was limited use of wheelchairs for all distances (ranging from 9.1% to 14.3%). Levels IV and V on the GMFCS had less variation in FMS scores and most of the children in these levels either did not complete the distances (no functional mobility at all distances) or used a wheelchair for mobility.

**Discussion:**

The Arabic FMS was shown to be a reliable and valid measure of functional mobility for children with CP in their environment based on the parental reports. Functional mobility varied at different distances and within each GMFCS level. The use of both the GMFCS and FMS when assessing children with CP is recommended.

## 1. Introduction

Cerebral palsy (CP) is a group of lifelong disorders that may cause activity limitation and abnormal posture. CP is common in Arab-speaking countries, with prevalence rates of 1.8/1,000 and 2.34/1,000 in the Arab world and Saudi Arabia, respectively (1, 2). Frequently, children with CP have limited functional mobility (3), which is a crucial component of the activity and participation domains in Part 1 of the International Classification of Functioning, Disability and Health (ICF), developed by the World Health Organization (4). However, clinicians and researchers are not always able to observe and measure functional mobility for children with CP in their settings. The availability of a valid and reliable, self-or parent-reported outcome measure for assessing functional mobility and determining changes over time and after an intervention is essential (5).

Currently, only a few tools are available for measuring the functional mobility for children with CP. In comparing these tools, the functional mobility scale (FMS) is the only tool which describes the use of different types of assistive devices while moving within different distances (6). The FMS is a valid and reliable outcome measure designed to evaluate functional mobility in children and adolescents with CP aged 4–18 years (5, 7–9). It is a self- or parent-reported outcome measure that is administered by a clinician in a semi-structured interview. It consists of six levels based on the usual need for assistive devices (such as walkers, crutches, or wheelchairs) required for walking or moving in three environments; home, school, and community (5 m, 50 m, and 500 m respectively) (7).

Self-report measures are crucial for clinical assessment and research as they allow for the assessment of the patient’s actual performance in his/her natural environment. However, these self-report measures need to be in the client’s native language to allow for valid responses. In addition, translating these measures in different languages would facilitate the collection of reliable data for research conducted in various countries and comparisons of the outcomes (10). Translation and cultural adaptation of the FMS has been performed for several languages such as Portuguese, Japanese and Greek (11–13); however, to our knowledge, an Arabic translation of the FMS has not been performed yet. Arabic is the official language in 22 countries with 456 million Arabic speakers around the world (14). To this extent, having an Arabic version of this measure would be of great benefit for children with CP, their families, researchers, and the treating clinicians around the Arab world. Therefore, the main objective of this research was to translate and culturally adapt the FMS into Arabic and to determine the psychometric properties of the translated version. In addition, the study also sought to explore functional mobility for children and adolescents with CP in Saudi Arabia.

## 2. Materials and methods

### 2.1. Design

This cross-sectional study was conducted over two phases: phase one included the translation of the original English FMS into Arabic; phase two involved examining the test–retest reliability and concurrent validity of the Arabic-translated version of the FMS.

### 2.2. Measures

#### 2.2.1. Family and child information form

This research was a part of a larger study exploring the characteristics of children with CP in Saudi Arabia. A self-report survey was used to collect demographic information regarding the child’s age, gender, and general health, as well as the parent’s age, level of education, work status, and family income.

#### 2.2.2. Functional mobility scale (FMS)

The FMS is a parent-report measure of a child’s mobility in three natural settings other than the clinical setting (home-5 m, school-50 m, and community-500 m) (7). Ratings are based on the use of assistive devices at these distances (3, 7), with scores ranging from 1 (using a wheelchair) to 6 (walking independently on all surfaces). The rating (C) indicates that the child crawls the 5-m distance, whereas the rating (N) means not applicable (the child has no functional mobility or does not finish the distance) (7). The inter-rater reliability and construct validity have been established for the FMS (3, 5, 9). Furthermore, it has been demonstrated that the FMS has the ability to differentiate between children with varying degrees of functional mobility and to detect change after any intervention (5, 7–9).

#### 2.2.3. Gross motor function classification system (GMFCS)

The GMFCS assesses a child’s current motor function capacity and limitations (15, 16). Level I indicates minimal limitations in gross motor function while Level V indicates maximal limitations and dependence in mobility skills. The GMFCS has evidence of content, construct, discriminative validity, and inter-rater reliability (15, 16). Both the FMS and the GMFCS examine the same construct, functional mobility, but have slight important differences. The FMS is a measure of a child’s performance and changes in his/her functional mobility over time (7). However, the GMFCS is a classification system of the child’s gross motor function, which is expected to be stable over time (17). The GMFCS levels of the participating children were determined by criterion-tested therapists.

### 2.3. Translation of the FMS into Arabic

**Figure 1:**
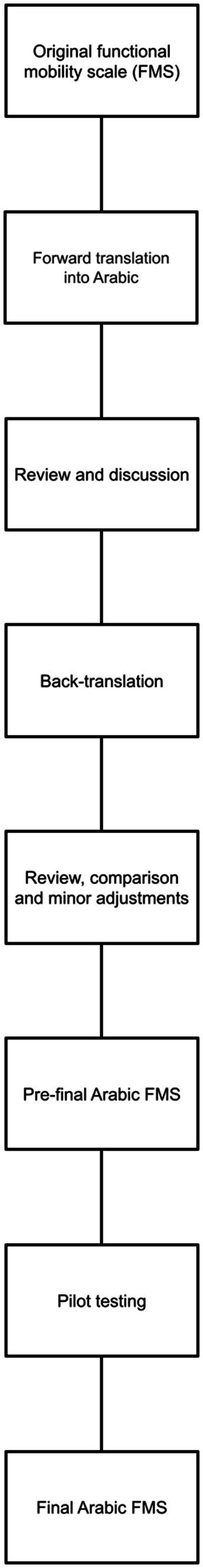
Steps of translating and cross-cultural adaptation of FMS into Arabic.

Permission from the publisher of the FMS was obtained to translate the scale into Arabic (“Obtained, with permission, from resources at The Royal Children’s Hospital, Melbourne, Australia https://www.rch.org.au/clinicalguide”). The translation methodology complied with the World Health Organization Disability Assessment Schedule 2.0 translation package (18), as shown in Figure 1. We formed a team of two researchers who are physical therapists (PTs) with experience in instrument development and translation, and one research assistant who is also a physical therapist. The translation and cultural adaptation process was achieved through six stages. First, one research assistant who is fluent in English and whose first language is Arabic, with 5 years of experience in physical therapy translated the original FMS. Second, the team reviewed and discussed the translated version to ensure that natural and culturally acceptable language was adopted. Third, the forward-translated version was given to an independent English-native translator who was unaware of the backward translation scale. Fourth, the original, the forward-translated, and the backward-translated versions were then sent to three Arab experts, who are PTs with PhDs, for evaluating the translation and determining its cultural appropriateness. They gave their approval with recommendations for minor adjustments such as choosing the appropriate Arabic name for each walking aid. One example is “walker,” the Arabic translation of it is not usually used by both parents and health care specialists, instead it is called by its English name “walker.” Therefore, our decision was to write both names in Arabic letters to help make it clearer. The pre-final version of FMS was then produced and used in a pilot study of 3 families with children diagnosed with CP. Feedback was received from the conducting physical therapists as well as the families, and no adjustments or clarifications were needed. Therefore, the Arabic version of the FMS was finalized.

### 2.4. Participants

Tables 1, 2 show the participants’ characteristics. A total of 154 participants were recruited (mean age 8.16 ± 3.32 years). We used a convenience sampling approach from 11 rehabilitation centers and hospitals that provided services to children with CP in 14 cities (representing the five different geographical areas) in Saudi Arabia. In other words, the chosen subjects for this study comprised children who were medically diagnosed with CP and were receiving services from public or private centers and hospitals at the time. Similarly, all participants’ parents read and signed an informed consent form prior to participation in the study. The study protocol followed ethical approval criteria according to the rules and regulations of the National Committee of Bioethics (NCBE) with approval from the local Ethics Committee of the University of Tabuk (UT-175-39-2022).

**Table 1:** Children’s characteristics (*N* = 154).

Children’s age-groups (years)	*N* (%)
Preschoolers (4–6)	53 (34.4)
School age (> 6–12)	84 (54.5)
Adolescents (>12–18)	17 (11.0)
Gender	
Male	94 (61.0)
Female	60 (39.0)

**Table 2:** Distribution of types of cerebral palsy by GMFCS levels.

Type of cerebral palsy	GMFCS					
	Level I	Level II	Level III	Level IV	Level V	Total
Spastic diplegia	5	19	20	10	2	56
Spastic hemiplegia	3	12	0	1	0	16
Spastic monoplegia	2	2	0	0	0	4
Spastic quadriplegia	0	4	2	10	31	47
Spastic triplegia	0	2	1	2	2	7
Other^a^	3	4	5	8	4	24
Total	13	43	28	31	39	154

GMFCS, Gross Motor Function Classification System.

a Ataxic, dyskinetic, hypotonic, mixed, and unknown.

### 2.5. Procedure

Twelve research assistants (RAs) who are physical therapists completed a two-hour training session to explain the study procedures and measures before starting data collection. Parents who visited the rehabilitation centers and hospitals for their child’s regular physical therapy appointments were invited to participate in the study. Upon agreement to participate, the RAs explained to the parents the aims, procedure, and expected outcomes of the study and obtained written informed consent. Parents were interviewed in two sessions. In session 1, the baseline assessment, the RAs interviewed the participating parents and completed the child and family information form. Then, parents were asked to rate the usual walking ability of their child on the Arabic FMS. Finally, RAs determined the child’s GMFCS level. In session 2, the re-test assessment, a sub-group of parents (*n* = 34) was asked to rate their child on Arabic FMS again. The mean time interval between the first and second sessions was 14.3 days (SD = 8.5), with a range of 6–37 days. The time interval between the two interviews was appropriate to leave enough time for the participants to forget their prior answers in the first session, but not too long for the children’s scores to change due to time.

### 2.6. Statistical analysis

Data was analyzed using SPSS Version 25. First, descriptive statistics and frequency tables were obtained. Concurrent validity was explored using Spearman’s rank correlation coefficient between scores of the Arabic FMS with their corresponding score on the GMFCS. Test–retest reliability was examined by conducting Cohen’s weighted kappa using data from 34 families who were re-interviewed to complete the Arabic FMS a second time.

## 3. Results

Almost half of the participating children were in a school-age group, and there were more males than females (Table 1). In addition, children were distributed on the five levels of GMFCS (Table 2), however, almost half of the sample were in the levels IV and V (Table 2).

### 3.1. Functional mobility in children with CP in Saudi Arabia

Table 3 describes FMS levels for children in relation to the three distances (5 m, 50 m, and 500 m). For the 5-meter distance, 33.7% of the children were independent walkers (FMS score 5 and 6), and for the 500 m, 23.3% were independent. Furthermore, functional mobility had greater limitation at the longer distances as the number of children who did not complete the distance (FMS score *N*) increased from 27.3% of children at 5 m to 55.2% at 500 m. Overall, there was limited use of wheelchairs ranging from 9.1% to 14.3%.

**Table 3:** Distribution of the FMS scores by distance.

Distance (m)	FMS score (*N* = 154)							
	1	2	3	4	5	6	C	N
5	14 (9.1%)	11 (7.1%)	2 (1.3%)	5 (3.2%)	38 (24.7%)	14 (9.1%)	28 (18.2%)	42 (27.3%)
50	19 (12.3%)	19 (12.3%)	1 (0.6%)	1 (0.6%)	37 (24.0%)	8 (5.2%)	0	69 (44.9%)
500	22 (14.3%)	7 (4.5%)	2 (1.3%)	2 (1.3%)	29 (18.8%)	7 (4.5%)	0	85 (55.2%)

Ratings: 6 – independent on all surfaces; 5 – independent on level surfaces; 4 – uses sticks (one or two); 3 – uses crutches; 2 – uses a walker or frame; 1 – uses wheelchair; C, crawling; N, does not apply, e.g., child does not complete the distance.

Table 4 shows the GMFCS levels, functional mobility varied within each level. In general, children in levels I and II (classified as highly functioning) were more independent walkers. However, children in level II had more variability in FMS scores at the three distances, from being an independent walker to a user of walkers and wheelchairs. Likewise, level III included variable functional mobility scores. On the other hand, levels IV and V had less variation in FMS scores and most of the children in these levels either did not complete the distances (no functional mobility in all distances) or used a wheelchair for mobility. Crawlers in 5 m distances were mostly level III and IV.

**Table 4:** Distribution of the FMS scores by the GMFCS levels at each distance.

GMFCS level	Distance (m)	FMS score							
		1	2	3	4	5	6	C	N
Level I (n)	5	0	0	0	0	5	8	0	0
	50	0	0	0	0	8	5	0	0
	500	1	0	0	0	8	4	0	0
Level II (*n*)	5	0	3	1	0	32	6	0	1
	50	1	6	1	0	29	3	0	3
	500	7	3	1	2	21	3	0	6
Level III	5	5	8	1	5	1	0	6	2
	50	8	11	0	1	0	0	0	8
	500	9	4	1	0	0	0	0	14
Level IV	5	6	0	0	0	0	0	19	6
	50	8	2	0	0	0	0	0	21
	500	3	0	0	0	0	0	0	28
Level V	5	3	0	0	0	0	0	3	33
	50	2	0	0	0	0	0	0	37
	500	2	0	0	0	0	0	0	37

FMS, Functional Mobility Scale; GMFCS, Gross Motor Function Classification System.

### 3.2. Concurrent validity

Table 5 shows Spearman’s *r* values between the Arabic FMS scores and the GMFCS scores with a range of (−0.895 and **–**0.779), indicating strong to very strong correlations (19). Negative values indicate an inverse relationship between the two scales. This is because higher FMS scores indicate better mobility, while lower GMFCS levels indicate better motor function.

**Table 5:** Spearman’s correlations between FMS and GMFCS scores.

Distance (m)	Correlation coefficient*
FMS 5	−0.895
FMS 50	−0.842
FMS 500	−0.779

*p < 0.01.

### 3.3. Test–retest reliability

For the test–retest reliability (Table 6), Kappa values showed almost perfect agreements as suggested by Landis and Koch (20), 0 = poor; 0.01–0.20 = slight; 0.21–0.40 = fair; 0.41–0.60 = moderate; 0.61–0.80 = substantial and 0.81–1 = almost perfect agreement (20).

**Table 6:** Test–retest reliability using Cohen’s weighted kappa (κ_w_).

Distance	κ_w_*	Agreement (%)
FMS-5	0.85 (0.75, 0.94)	67.6
FMS-50	0.95 (0.90, 0.99)	88.2
FMS-500	0.86 (0.73, 0.99)	85.3

*p < 0.01.

## 4. Discussion

This was the first study to explore functional mobility in children and adolescents with CP in Saudi Arabia. The first aim of the study was attained. The Arabic FMS was found to be valid and reliable as a means of assessing functional mobility in children with CP in the Arab culture. The concurrent validity was confirmed by the strong to very strong correlations between GMFCS scores and the Arabic FMS scores. Correlations in our study were slightly higher than those revealed in the Japanese study (*r*^2^ = −0.71 to −0.75) (12), but almost the same as those in the Greek study (*r*^2^ = −0.85 to **–**0.89) (13). In the Japanese study, they only included children with GMFCS levels I-II, which may explain the slight differences with our results. The test–retest reliability was also established, and thus demonstrating the stability of the FMS scores when the mobility status of the child does not change. It was also comparable with the previous studies as the Japanese study showed substantial to excellent level of agreement (kappa =0.72–0.87) (12), and almost perfect agreement for the Greek study (kappa = 96.6–100) (13). Our sample covered the 5 different geographical areas in Saudi Arabia, which is expected to be representative of children with CP in the country. The FMS was developed to be administered by clinicians through parent interviews, therefore the educational level of parents would not affect the scoring, as they would have the chance to ask for clarifications during the interview. Furthermore, the Arabic FMS (as the English one) has simple language with clear instructions and photos that a layperson can interpret and give the correct score.

The cross-cultural validation of the Arabic FMS is also established (at least in part) as parents answered questions regarding the three distances without any difficulties. This implies that the three distances correlate with Saudi homes, school environments and community settings. Mobility ratings were also used by parents, indicating that the range of functional mobility scores represents children with CP in Saudi Arabia.

The availability of a scale that assesses a child’s mobility in his/her natural environment is very crucial. Children with CP were shown to differ in their mobility across environmental settings (21) and thus, clinicians need to examine the performance of children in environments that are important to the child’s daily life. Having a reliable and valid Arabic version of the FMS will help clinicians to evaluate a child’s actual performance based on parental reports in their native language. The original FMS was constructed to assess mobility and change in mobility as the child grows or after intervention (15). We recommend further research to assess the responsiveness of the Arabic FMS.

The second aim of the study was to explore functional mobility in children and adolescents with CP in Saudi Arabia. Functional mobility was found to be limited in children with severe CP (GMFCS IV and V). Likewise, there was greater limitation of mobility at longer distances. This should be of concern for clinicians as limited mobility may lead to complications such as joint contractures, scoliosis, pain, as well as decreased participation and quality of life (22, 23).

It is interesting to find out that functional mobility varied within each GMFCS level. This implies that children may have the same GMFCS classification but differ in their functional mobility. Rethlefsen et al. (24) have shown similar results where children with CP of the same GMFCS level varied in their mobility, with the most variability in levels II and III. This variability can be related to personal preferences, environmental constraints, or age (22). Therefore, it is recommended when assessing a child with CP to consider both the GMFCS and FMS (5 m, 50 m, and 500 m).

The high percentage of children with no functional mobility (rated N) indicates that these children do not complete the specified distance neither when walking nor in a wheelchair. They are most probably carried by caregivers and would most likely have problems accessing school and community settings. Our results are comparable to the results of the previous study of Saleh et al. (25), which was done in Jordan (neighboring country), where 32% and 50% of all children had no form of functional mobility at school or in the community (*N* rating). Saleh et al. included children from all GMFCS levels and almost half of their sample were categorized as being within the most severe levels of GMFCS (IV and V). This is similar to our sample and may explain similarities in the findings. Other studies included children in GMFCS levels II–IV (5) or I–III (15), which corresponds to children with greater functional levels. Thus, it is expected that less children would have the FMS score of (*N*).

It is important to note that limited functional mobility at long distances such as in school and community settings may be related to the environment itself or to the lack of adaptive equipment or assistive devices (26). However, in Saudi Arabia, all government and private buildings including schools, hospitals, shopping centers and other community settings are required by law to be accessible for people with disabilities. Therefore, further research is warranted to explore reasons for limited functional mobility in children with CP at long distances in Saudi Arabia.

### 4.1. Limitations

The FMS was translated into classic Arabic, the formal and standard type of Arabic used in all Arab countries. However, further studies may be needed to evaluate its validity in other Arab cultures.

## 5. Conclusion

The Arabic FMS was shown to be a reliable and valid measure of functional mobility for children with CP. It is a simple and practical outcome measure that will help clinicians to assess children’s performance in their environment based on parental reports. Functional mobility varied between distances and within each GMFCS level. The use of both the GMFCS and FMS when assessing children with CP will give more comprehensive information about the child’s function and is therefore recommended.

## Data availability statement

The datasets presented in this article are not readily available because database is only accessible to authors as stated to participants in the informed consent. Requests to access the datasets should be directed to aa-albalwi@ut.edu.sa.

## Ethics statement

The studies involving human participants were reviewed and approved by local Ethics Committee of the University of Tabuk (UT-175-39-2022). Written informed consent to participate in this study was provided by the participants’ legal guardian/next of kin.

## Author contributions

AbA and MS proposed the study, developed the survey, organized the database, statistical analysis, and wrote the manuscript. AhA served in the study proposal, organized the database, and writing manuscript. QA contributed to statistical analysis and writing manuscript. SA contributed to organize the database, reviewed and edited the manuscript. All authors contributed to the article and approved it for publication.

## Funding

This study received funding from the Deanship of Scientific Research at the University of Tabuk research no. (S-1442-0166).

## Conflict of interest

The authors declare that the research was conducted in the absence of any commercial or financial relationships that could be construed as a potential conflict of interest.

## Publisher’s note

All claims expressed in this article are solely those of the authors and do not necessarily represent those of their affiliated organizations, or those of the publisher, the editors and the reviewers. Any product that may be evaluated in this article, or claim that may be made by its manufacturer, is not guaranteed or endorsed by the publisher.
